# Life Lengthened

**Published:** 1867-12

**Authors:** 


LIFE LENGTHENED.—In all countries and all latitudes, the well-to-do live longer than the poor by an average of eleven years; this shows the deleterious influence of an anxious mind on the bodily health, the anxiety for to morrow's bread. Pensioned persons live indefinitely long; the poor-houses of Great Britain can any day turn out a large army of men and women among the eighties and nineties who have been in those institutions for twenty and thirty years, owing in great part to an habitual feeling of confidence that ample provision is made for the future, and the mind is at rest; but it must not be forgotten that the cleanliness, the plain food and the regular habits, compulsory in those institutions contribute greatly to the same end. Insurance companies are ingeniously making use of these facts, and doubtless with some show of reason, for certainly a man feels greatly more at ease in his mind when he feels assured that in case of his death, a handsome amount of money would certainly be paid over to his family, than if the blighting and depressing impression were all the time weighing upon his spirits that his death would leave them in destitution. A British medical journal says that in the eighteenth annual report of the Providential Assurance Co., it is shown that for three years in succession, the rate of mortality was twenty-three per thousand among the unassured, while it was twenty-one per thousand among the same class and occupation of persons who kept up their assurance. It is beyond all question that a quiet mind contributes to the health and well-being of the body. In this connection it may be stated that

